# Effect of PEEP on lung aeration in pediatric patients after cardiac surgery: a CT-Based study

**DOI:** 10.1016/j.bjane.2025.844623

**Published:** 2025-04-22

**Authors:** Solange C. Gimenez, Milene C. Carrilho, Isabela M. Malbouisson, Marcelo Gama de Abreu, Jean-Jacques Rouby, Luiz Marcelo Sá Malbouisson

**Affiliations:** a Hospital das Clínicas da Faculdade de Medicina da Universidade de São Paulo, Divisão de Anestesia, São Paulo, SP, Brazil; b University Paris-Sorbonne (UPMC Paris 06), Hôpital de la Pitié-Salpêtrière, Assistance Publique Hôpitaux de Paris, Multidisciplinary Intensive Care Unit, Department of Anesthesiology, Paris, France

**Keywords:** Cardiopulmonary bypass, Congenital heart defects, Positive end-expiratory pressure, Pulmonary atelectasis, Tomography

## Abstract

**Background:**

Loss of lung aeration is frequently observed in adult patients following cardiac surgery with cardiopulmonary bypass. Yet, in children, changes in lung aeration following surgical repair of congenital heart defects, and the effects of Positive End-Expiratory Pressure (PEEP), remain uncertain.

**Methods:**

Changes in lung aeration were investigated using volumetric computed tomography in 12 children with congenital acianogenic heart diseases and increased pulmonary flow who underwent total surgical repair under cardiopulmonary bypass. Computed tomography of the lungs was obtained preoperatively during spontaneous breathing and postoperatively during mechanical ventilation with positive end-expiratory pressure of 0, 5 and 10 cm H_2_O. Gas and tissue lung volume and mass, as well non-aerated, poorly aerated and normally aerated lung compartments were measured.

**Results:**

Median age of patients was 18.3 months, (4 to 24 months), weight was 9.3 ± 2.3 kg. Cardiopulmonary bypass duration was 77 ± 26 minutes. Preoperatively, pulmonary volume was 545 mL (237‒753 mL), whereby tissue and gas volumes were 48.4% (41.7%‒59.6%), and 51.6% (40.4%‒58.3%), respectively. Non-aerated and normally aerated compartments accounted for 15% and 47.9% of lung tissue, respectively. Postoperatively, at zero PEEP, the non-aerated compartment increased to 27%, while normally-aerated compared decreased to 38.5%. Stepwise PEEP application restored normally aerated lung volume to preoperative levels but did not significantly reduce non-aerated parenchyma.

**Conclusion:**

Loss of lung aeration was pronounced after surgical correction of congenital heart defects. PEEP up to 10 cm H_2_O restored gas volume but failed to recruit the collapsed parenchyma. Ethical Approval CAPPesq n° 854/01.

## Introduction

Atelectasis formation and increased Extravascular Lung Water (EVLW) due to CPB-induced inflammation are common in patients undergoing cardiac surgery.^1^ In adults, pulmonary alterations are almost universally present.^2^ Several mechanisms have been implicated: a) An up-ward shift of relaxed diaphragm induced by abdominal weight contents;^3^ b) Cardiac-induced compression of the dependent lower lobes^4^ and c) Pulmonary inflammation promoting increase in EVLW and lung weight causing compression of the dependent lung regions^5^ and alterations of surfactant properties.^6^

Contrasting with numerous studies performed in adults, few information is available in pediatric patients. In this population, two additional factors can further contribute to postoperative pulmonary dysfunction: 1) Marked enlargement of the heart, compressing bronchi and underneath lung and decreasing lower lobe’s ventilation,^7^ and 2) Pulmonary edema resulting from intra-cardiac shunt.

This study investigates post-surgical lung aeration changes in children with congenital heart defects and assesses the effect of increasing Positive End-Expiratory Pressure (PEEP) on pulmonary aeration using Computed Tomography (CT).

## Material and methods

### Study design and patients

This pre-post observational study with an intervention component was performed at the Heart Institute (InCor) of Hospital da Clínicas da Universidade de São Paulo and approved by the institutional ethics committee (CAPPesq n° 854/01). Patients’ relatives or legal guardians signed a free Informed consent form. Fourteen consecutive children with congenital heart disease with elective indication of surgical repair of their anatomical heart defects were selected for the study. This study was conducted as an academic research project without dedicated funding support.

Inclusion criteria were: 1) Hemodynamic stability characterized by absence of inotropic support before surgery; 2) Non-hypoxemic high pulmonary flow diagnosed by trans-thoracic echocardiography or cardiac catheterism with pulse oximetry greater than 95% in room air; 3) Age lower than 2-years. Exclusion criteria were 1) Untreated respiratory infections; 2) Overt pulmonary edema; 3) Necessity of oxygen supplementation; and 4) Incomplete surgical correction of the heart defect.

On the day before surgery, patients were transported to the Department of Radiology and a fast thoracic CT scan was obtained. Due to incapacity to obey commands and adequately hold the inspiration, children freely breathed during the preoperative CT acquisition. In order to ensure safety, the children were secured to CT scan tray table by means of auto adhesive tissue bands wrapping the top of the head, hip and lower extremities. A physician adequately protected against radiation stayed in the CT room during the entire acquisition. ECG, non-invasive arterial pressure and pulse oximetry were monitored during the acquisition using a Philips M3 transport monitor (Philips, Eindhoven, The Netherlands).

Heart surgical repairs were performed under hypothermic Cardiopulmonary Bypass (CPB) using equipment from Braile Biomédica (São José do Rio Preto, São Paulo, Brazil). General anesthesia was induced with sevoflurane inhalation in 100% oxygen to facilitate the placement of a peripheral venous catheter. Patients were intubated using a combination of sevoflurane, ketamine (1 mg.kg^-1^), fentanyl (5 µg.kg^-1^), and rocuronium (1 mg.kg^-1^). Anesthesia was maintained with sevoflurane, fentanyl, and pancuronium.

During CPB, an additional dose of midazolam was administered to ensure adequate hypnosis and amnesia. Mechanical ventilation was initiated with the Primus Anesthesia Workstation (DRÄGER MEDICAL, Lübeck, Germany) in pressure-controlled ventilation mode. Inspiratory pressure was adjusted to maintain a tidal volume of 8 mL.kg^-1^, and the respiratory rate was regulated to achieve PaCO_2_ values between 35 and 45 mmHg, avoiding auto-PEEP. Inspiratory time was adjusted to 33% of the respiratory cycle, with an FiO_2_ of 40% and a PEEP of 5 cmH_2_O.

At the conclusion of CPB, blood ultrafiltration was performed to remove excess fluid and achieve a zero CPB fluid balance. Postoperatively, patients were sedated with fentanyl and midazolam until the following morning to ensure complete clinical stabilization. Ventilation was continued with a Galileo ventilator (Hamilton Medical AG, Bonaduz, Switzerland), following the same intraoperative ventilation strategy.

In the morning of first postoperative day, thoracic CT images were obtained during a 30 second expiratory pause in 3 conditions: zero end-expiratory pressure (zero PEEP), 5 and 10 cm H_2_O of PEEP. Each CT scan was preceded by 10 minutes of ventilation at the designated PEEP level to ensure lung stabilization. No alveolar recruitment maneuvers were applied during the CT acquisition. No complications occurred during transport and in the CT facility. Postoperative weight was measured in the ICU before transportation to the Department of Radiology.

### Acquisition of the CT images

**Figure 1:**
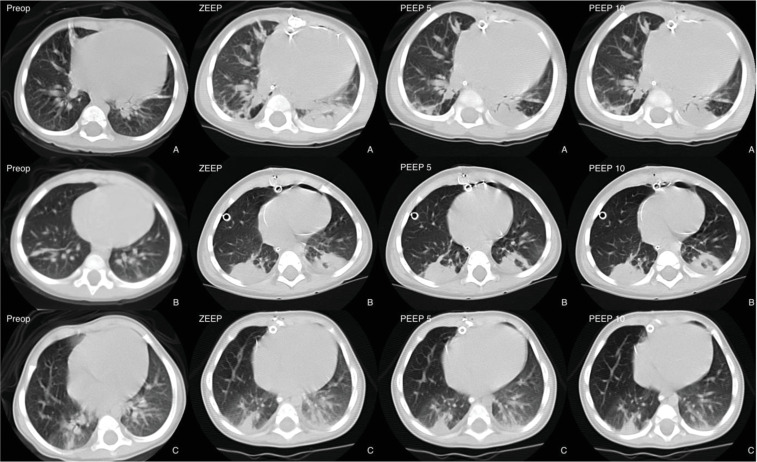
Representative CT images obtained 1 cm above the diaphragm in the preoperative (Preop) and postoperative periods in zero PEEP conditions (zero PEEP), 5 cm H_2_O PEEP (PEEP 5) and 10 cm H_2_O PEEP (PEEP 10) in three patients (A, B and C).

The methods used to acquire and analysis the lung CT images were described previously.^4^^,^^8^^,^^9^ Briefly, lung images were obtaining using a Toshiba Aquilion 16 CT scanner (Toshiba Medical Division, Japan), with exposures taken at 80 kV and 125 mAs. The thoracic images were reconstructed as contiguous 5-mm thick axial sections from the volumetric data. Figure 1 shows representative images of CT images obtained preoperatively and postoperatively at zero PEEP, 5 and 10 cm H_2_O of PEEP in 3 patients.

Volumetric CT images were analyzed as follows: initially, we delineated the external boundaries of both left and right lung parenchyma in each thoracic CT image using the Osiris software (Geneve University Hospital, Geneve, Switzerland). In each delineated region, the pixels contained were distributed in 1200 compartments in an electronic spreadsheet, according to their X-Ray attenuation coefficient (CT number). Using the equations presented below, it is possible to compute the mass, tissue volume and gas volume with a given delineated lung region in a thoracic CT image:

1. Volume of the voxel = (size of the pixel)^2^ × section thickness
2. Total volume = number of voxels × volume of the voxel
3. Volume of gas = (- CT number/1000) × Total volume, if the compartment considered has a CT number below 0 (volume of gas = 0 if the compartment considered has a CT number above 0).
4. Volume of tissue = (1 + CT number/1000) × Total volume, if the compartment considered has a CT number below 0, or (4’) Volume of tissue = number of voxels × volume of the voxel, if the compartment considered has a CT number above 0.
5. Mass of lung tissue = volume of lung tissue if the compartment considered has a CT number greater than 0, or (5’) Weight of lung tissue = (1 + CT number/1000) × volume of lung tissue if the compartment considered has a CT number below 0.

The overall lung tissue mass and pulmonary were computed by adding all of the partial masses and volumes on the thoracic CT image. The tissue mass calculation is possible due to an almost linear correlation between CT number (from -1000 to +100 HU) and physical density.^10^

The degree of pulmonary aeration was categorized using the following criteria: 1) Hyperaerated lung parenchyma (CT numbers from -1000 HU to -900 HU);^11^ 2) Normally aerated lung parenchyma (CT numbers from -900 HU to -500 HU);^11^ 3) Poorly aerated lung parenchyma (CT numbers from -500 HU to -100 HU);^11^ and 4) Non-aerated lung parenchyma (CT numbers from -100 to +100 HU).^12^

### Statistical analysis

A convenience sample of 14 patients, deemed representative, was selected to minimize unnecessary radiation exposure from computed tomography. This approach ensured the study's feasibility while prioritizing patient safety in the absence of comparable prior research.

Normal distribution of data was evaluated by means of visual assessment and Shapiro-Wilk test. Comparison between pre and postoperative weight was performed using Wilcoxon test. Volumetric and weight data obtained from CT analysis in the preoperative period and in the postoperative period at 0, 5 and 10 cm H_2_O PEEP were compared by means of Friedman test, followed by Dunn post-hoc test when indicated. In order to enable comparison among children of different age and size, the different CT volumes obtained in 0, 5 and 10 cm H_2_O PEEP were expressed as percentage of the overall lung volume measured in each condition. Comparisons between the left and right lung volumes were obtained by means of Mann-Whitney test or unpaired Student *t*-test. Correlations between CT scan and physiological data were performed by linear regression analysis or Spearman rank order analysis. All data were presented as mean ± SD, median (minimum – maximum) or as specified otherwise. The significance level was fixed at 5%. Statistical analysis were performed using Stata 12 software (StataCorp, College Station, TX, USA).

## Results

### Study population

Two patients of the 14 initially selected for inclusion were excluded. One patient presented pulmonary edema and the second was excluded due to technical difficulties for recording CT images. Twelve patients completed the study, of whom three had isolated ventricular septal defects, and three had ventricular septal defects associated with a patent ductus arteriosus. Two patients presented with atrial septal defects, two were diagnosed with complete atrioventricular canal defects, one had partial anomalous pulmonary venous connection, and one had partial atrioventricular canal defects. Characteristics of the 12 patients enrolled in the study are presented in Table 1. The mean age of the children was 18.3 months, ranging from 4 to 24 months. The mean preoperative weight was 9.3 ± 2.3 kg. Duration of surgery was 420 ± 47 minutes, CPB duration was 77 ± 26 minutes, and the aortic cross-clamping time was 50 ± 27 minutes. All patients achieved hemodynamic stability in immediate postoperative period, allowing weaning of inotropic drugs within the first 36 hours. Mean postoperative weight was 9.63 ± 2.26 kg, which represented a 3.1% increase when compared to preoperative values (p < 0.001). All patients were discharged from the ICU without postoperative complications, the ICU length of stay ranging from 48 to 93 hours.

**Table 1:** Demographic and surgical characteristics of the patients.

Patient	Age (months)	Height (cm)	Preoperative Weight (g)	Postoperative Weight (g)	Diagnosis	CPB (min)	Predicted FRC*
1	16	70	8410	8450	PAVCD	105	0.38
2	24	88	10900	11000	VSD + PDA	72	0.26
3	15	80	10640	10730	VSD	60	0.22
4	12	86	9900	10000	PAPVC	82	0.30
5	24	85	10500	10650	TAVCD	126	0.45
6	22	70	8400	8800	ASD	53	0.19
7	17	98	12000	12500	VSD	90	0.32
8	24	87	12000	12030	ASD	45	0.16
9	20	74.5	9700	9900	VSD	90	0.32
10	9	55	5585	6100	VSD + PDA	50	0.18
11	4	60	4535	4900	VSD + PDA	53	0.19
12	24	85.5	10030	10500	TAVCD	105	0.38

CPB, Cardiopulmonary Bypass; ASD, Atrial Septal Defect; VSD, Ventricular Septal Defect; PDA, Patent Ductus Arteriosus; TAVCD, Total Atrioventricular Canal Defects, PAVCD, Partial Atrioventricular Canal Defects; PAPVC, Partial Anomalous Pulmonary Venosus Connection; FRC*, Functional Residual Capacity calculated according to the equation proposed by Stocks and Quanjer.

### Preoperative CT assessment of lung volumes and weights

**Figure 2:**
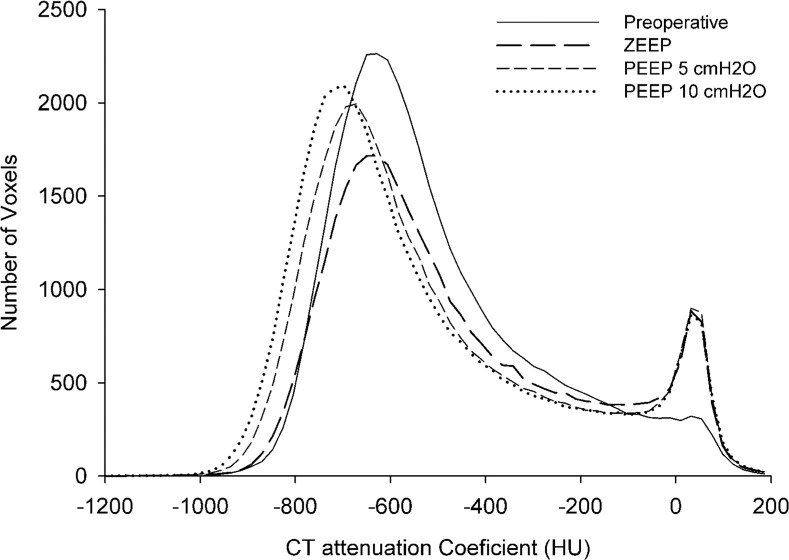
Frequency distribution of pulmonary radiological densities expressed as CT attenuation coefficient observed in the preoperative CT acquisition (solid line), postoperatively in zero PEEP condition (long bold dashed line), in 5 cm H_2_O PEEP condition (short, dashed line) and in 10 cm H_2_O PEEP condition (dotted line).

Figure 2 presents the mean CT attenuation histograms from whole-lung CT scans obtained preoperatively and postoperatively under zero PEEP, 5 cm H_2_O PEEP, and 10 cm H_2_O PEEP conditions. Preoperatively, the median total pulmonary volume was 545 mL (237‒753 mL), with a median lung weight of 253 g (134‒331 g). When normalized to total body weight, lung weight accounted for 2.8% (1.8%‒3.6%). The right lung contributed 55.6% ± 4.4% of the total lung volume, while the left lung made up 44.4% ± 4.4%. Tissue volume constituted 48.4% (41.7%‒59.6%) of the total lung volume, while gas volume accounted for 51.6% (40.4%‒58.3%).

Regarding lung aeration compartments, non-aerated lung parenchyma represented 15% (9.7%‒17.2%) of the total lung weight, poorly aerated lung parenchyma accounted for 36% (28.8%‒60%), normally aerated lung parenchyma made up 47.9% (24.7%‒59.1%), and hyperaerated parenchyma remained minimal (< 0.1% of the total lung). No significant differences were observed between right and left lungs concerning total volume, tissue and gas volumes, weight, and lung parenchyma aeration distribution (Table 2).

**Table 2:** Preoperative overall, gas and tissue volumes and weight of right and left lungs and analysis of lung according to CT attenuation.

			Right lung		Left lung		p-value
Volumetric CT analysis	Volume	Overall volume (mL)	313.7	(14 ‒ 419)	233.5	(96 ‒ 334)	0.06
		Gas volume (%)	53.8	(40 ‒ 61)	47.6	(35 ‒ 55)	0.11
		Tissue volume (%)	46.2	(39 ‒ 61)	52.5	(45 ‒ 65)	0.11
	Tissue weight	Overall tissue (g)	139.3	(76 ‒ 184)	120.3	(59 ‒ 149)	0.17
Lung analysis according to CT attenuation	Percentage of lung weight	OIP (%)	0	(0 ‒ 0.1)	0	(0 ‒ 0.1)	‒
		NoIP (%)	51.5	(25 ‒ 64)	42.2	(14 ‒ 58)	0.29
		PIP (%)	33.8	(25 ‒ 60)	40.8	(31 ‒ 68)	0.14
		NIP (%)	13.7	(10 ‒18)	15.5	(9 ‒ 20)	0.23

OIP, Overinflated Parenchyma; NoIP, Normally-Inflated Parenchyma; PIP, Poorly-Inflated Parenchyma; NIP, Non-Inflated Parenchyma. Data are expressed as mean standard deviation. The variables were compared by means of a Mann-Whitney test.

### Impact of surgical intervention and PEEP on lung aeration and morphology

Following surgery, at zero PEEP, overall lung volume decreased by 6.6% compared to preoperative values. This reduction was primarily due to an 18.1% decrease in gas volume, while tissue volume and lung weight remained unchanged (Table 3). PEEP administration at 5 cm H_2_O restored gas volume to preoperative levels (277 mL), and at 10 cm H_2_O, gas volume increased further to 291 mL. Median lung weight and tissue volume remained stable across PEEP conditions. The gas-to-lung tissue weight ratio increased progressively from 0.9 mL.g^-1^ at zero PEEP to 1.0 mL.g^-1^ at PEEP 5 cm H_2_O and 1.1 mL.g^-1^ at PEEP 10 cm H_2_O.

**Table 3:** Overall, gas and tissue volume and weight of lungs CT scans obtained preoperatively and postoperatively at zero PEEP, 5 and 10 cm H_2_O PEEP.

		Preop		zero PEEP		PEEP 5		PEEP 10	
**Volume (mL)**	Overall volume	545	(237 ‒ 753)	501	(199 ‒ 608)^a^	512	(200 ‒ 660)	558	(205 ‒ 709)
	Gas volume	271	(103 ‒ 423)	222	(68 ‒ 316)^a^	277	(65 ‒387)	291	(72 ‒ 432)
	Tissue volume	253	(134 ‒ 330)	263	(131 ‒ 300)	255	(135 ‒ 341)	245	(133 ‒ 362)
**Tissue weight (g)**	Entire lungs	254	(135 ‒ 332)	265	(133 ‒ 303)	257	(137 ‒ 345)	247	(135 ‒ 366)

Preop, Preoperative; zero PEEP, Zero End-Expiratory Pressure; PEEP 5, Positive End-Expiratory Pressure of 5 cm H_2_O; PEEP 10, Positive End-Expiratory Pressure of 10 cm H_2_O; Data are expressed as median (minimum – maximum). The variables were compared by means of a Friedmann test.

a Means p < 0.05 when compared to preoperative values in pairwise multiple comparison.

**Figure 3:**
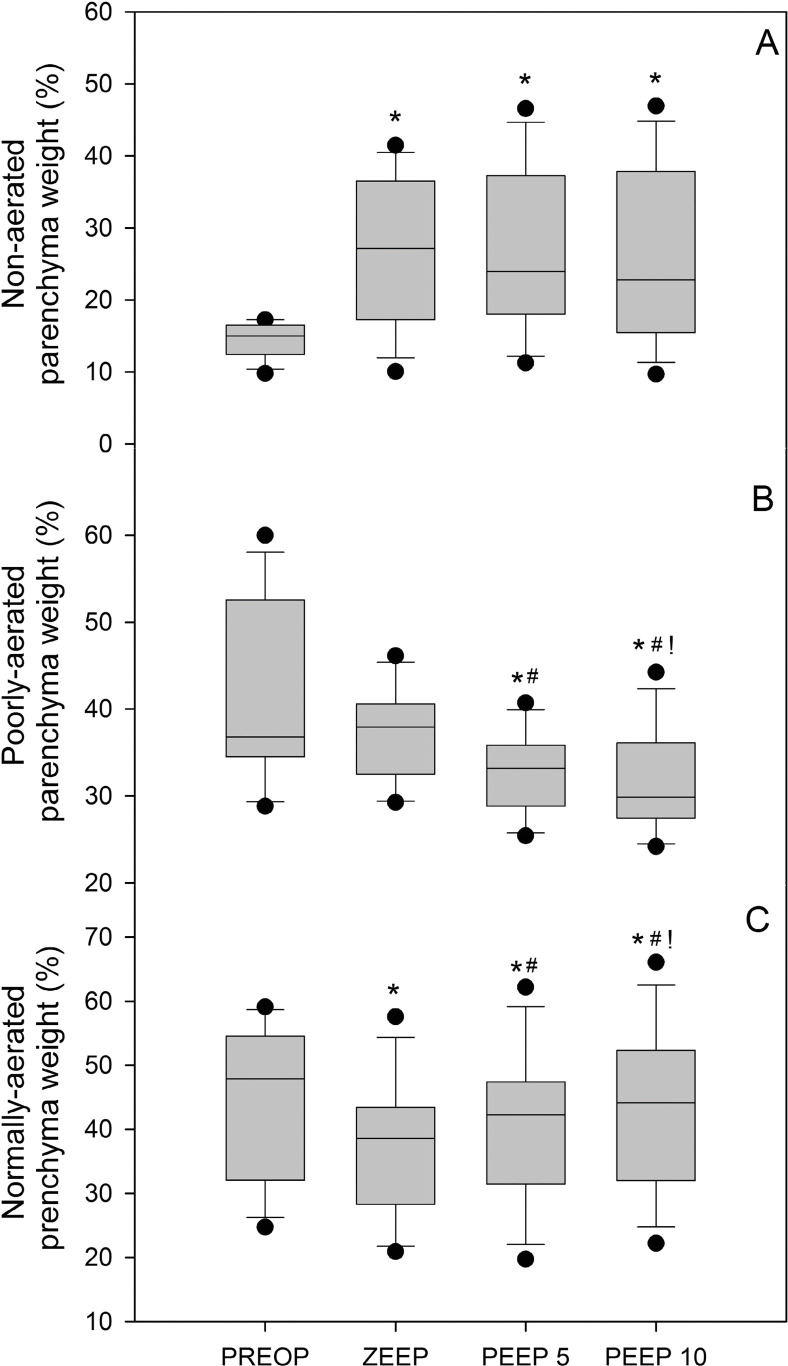
Percentage of non-aerated lung parenchyma weight (upper panel A), poorly aerated lung parenchyma weight (middle panel B) and normally-aerated lung parenchyma weight (lower panel C) computed preoperatively (PREOP) and in conditions of zero end-expiratory pressure (zero PEEP), 5 (PEEP 5) and 10 cm H_2_O of PEEP (PEEP 10) postoperatively. The symbol (*) means different from PREOP (p < 0.05), (#) means different from zero PEEP (p < 0.05) and (!) means different from PEEP 5 (p < 0.05).

As shown in Figure 3, surgery significantly increased the fraction of non-aerated lung parenchyma from 15% preoperatively to 27% postoperatively at zero PEEP. Notably, the application of PEEP at both 5 and 10 cm H_2_O failed to reduce the non-aerated parenchyma fraction. The fraction of normally aerated lung parenchyma significantly decreased from 47.9% preoperatively to 38.5% postoperatively at zero PEEP. PEEP administration progressively increased normally aerated lung fraction to 42.2% and 44.1% at PEEP 5 cm H_2_O and 10 cm H_2_O, respectively. Despite this improvement, PEEP was unable to fully restore normally aerated lung tissue to preoperative levels.

Poorly aerated lung parenchyma fraction remained unchanged postoperatively at zero PEEP but showed a significant and progressive reduction with PEEP administration. The fraction of hyperaerated lung parenchyma remained minimal (≤ 0.1%) across all conditions.

## Discussion

The main findings of the study are that, in pediatric patients undergoing total surgical repair under cardiopulmonary bypass: 1) Lung tissue volume did not change while overall gas volume decreased in the postoperative period at zero PEEP; 2) Postoperatively, there was an increase in poorly- and non-aerated lung parenchyma mass at zero PEEP; and 3) PEEP levels up to 10 cm H_2_O promoted an increment in the amount of normally-aerated lung parenchyma but did not decreased the amount of collapsed lung tissue.

### Methodological considerations

Spatial resolution influences computed tomography assessment of degree of lung aeration loss.^13^ In the present study, voxel volume was 0.06 ± 0.01 mL (CT section thickness = 0.5 mm, matrix 512 × 512 pixels and pixel size = 0.35 mm). Considering that fully developed alveoli are spherical-like structures with a radius of 0.1 mm at functional residual capacity,^14^ the alveolar volume is 0.0042 mL and a voxel would encompass about 14 alveoli. Such CT technical conditions by limiting partial volume effect, provide a high accuracy for measuring the different degrees of aeration loss. A few concerns must be kept in mind when interpreting pediatric volumetric CT data. Lung parenchyma growth process occurs until 8-years of age, a period during which the number of alveoli increases rapidly, as only one-third to one-half of alveoli characterizing the adult lung are present at birth.^15^^,^^16^ During the first two years of life, the gas exchanging open structures of the architecturally immature lung alveolar saccules undergo a septation process, giving place to respiratory bronchioles, alveolar ducts and sacs.^17^ After this first period, further lung growth occurs essentially by an increase in alveolar size and respiratory airway caliber.^18^ Children with ages ranging from 4 months to 24 months were enrolled in this study. We do not know whether voxels of equal volume observed in the same lung region in children of different ages could contain similar number of alveoli, gas or tissue. Unfortunately, it is very difficult to select children within a narrow range of age to avoid this source of bias while respecting strict inclusion criteria. Since median gas/tissue weight ratio remained close to 1 mL.g^-1^ in the preoperative period and did not present any significant relationship to aging, it can be reasonably assumed that voxels were composed of similar amounts of gas and tissue from 4-months to 2-years.

### Lung aeration in children with congenital heart disease

The gas and tissue distribution within the lungs of children suffering from left-to-right shunt congenital heart disease represented 51.6% and 48.4% of the overall lung volume, respectively, in preoperative CT scans, with a mean lung density of 1 g.mL^-1^. Additionally, 15% of lung parenchyma was non-aerated, 36% poorly aerated, and only 47.9% was normally aerated.

No previous study has evaluated the distribution of gas and tissue within healthy children’s lungs to enable direct comparisons. However, data from healthy adult volunteers by Puybasset et al. showed that gas and tissue volumes comprised 70% and 30% of overall lung volume, respectively.^9^ Vieira et al. reported that 92 ± 3% of overall lung parenchyma was normally aerated, 7 ± 3% was poorly aerated, and less than 1% was non-aerated.^11^ In children aged 7 to 25 months undergoing CT imaging for non-respiratory medical issues, Martínez et al. showed that pulmonary voxels were characterized by CT attenuation coefficients ranging from -200 HU to -800 HU, with a mean lung tissue density of 0.43.^19^

Physiologically, we would expect the gas fraction within healthy children’s lungs to be higher than in adults since their lungs are structurally less developed, with fewer septations and less alveolar tissue. However, our findings indicate the opposite. This discrepancy is likely due to the increased pulmonary blood volume in children with congenital heart disease. Left-to-right shunts result in increased pulmonary circulating blood volume, leading to enlarged pulmonary blood vessels and reducing available space for air. Furthermore, sustained high pulmonary blood flow causes thickening of the pulmonary arterial walls, further decreasing the gas fraction. These vascular changes result in higher lung tissue density and an abnormally elevated fraction of poorly and non-aerated parenchyma.

Although preoperative FRC measurements were obtained in spontaneous, uncontrolled breathing conditions and are not directly comparable to predicted FRC values calculated using validated standard formulas,^20^ median predicted FRC values (256 mL [91–395 mL]) were within the same range as preoperative CT gas volume (271 mL [103–423 mL]). Therefore, we can infer that pulmonary gas volume was not significantly affected by congenital heart disease. Instead, the observed increase in pulmonary tissue volume likely results from the high pulmonary blood flow characteristic of congenital cardiac disease. Left-to-right shunts increase pulmonary circulating blood volume, induce pulmonary arterial dilation, and recruit additional pulmonary capillary beds.^21^ The voxels in these vascular areas exhibit fluid densities close to water, explaining the elevated fraction of poorly and non-aerated parenchyma. Additionally, thickening of pulmonary arterial walls due to sustained pulmonary hypertension may contribute further to the increase in lung tissue.^22^

### Impact of anesthesia and surgery on postoperative lung aeration

As expected, the gas lung volume decrease in the postoperative zero PEEP CT. Different conditions of CT acquisition may partly account for this loss of aeration. In the preoperative period, CT sections were obtained in spontaneous breath, whereas CT sections were obtained during an expiratory pause in the postoperative period. Other factors, however, certainly played a major role in aeration loss. Atelectasis preferentially distributed in dependent and caudal regions, near the diaphragm, were found as previously observed in adult patients undergoing cardiac surgery with CPB.^22^ In the postoperative period using zero PEEP, non-aerated lung weight increased by 12%, normally aerated weight decreased from 47.9% to 38.5%, while no significant alterations were observed in the poorly aerated compartment. Probably, some normally aerated lung regions became poorly aerated while poorly-aerated lung regions became non-aerated. As in adults, loss of aeration observed in the children after cardiac surgery can be explained by: 1) CPB-induced surfactant activity reduction;^6^^,^^23^ 2) Lung parenchyma mechanical compression resulting from the upward shift of relaxed diaphragm into the thorax;^3^ 3) Surgical manipulation of intrathoracic structures; and 4) Compression of lower lobes by the heart. In this particular population, heart weight-induced lower lobe’s compression may critically contribute to pulmonary collapse. The heart is enlarged because of the fluid-overloaded hyperdynamic pulmonary circulation and lay over a greater part of lower lobes.^4^^,^^7^^,^^24^ The current data do not support the hypothesis that lower lobe compression is caused by the overlying edematous lung with increased extravascular lung water.^5^ Despite the well-known increase in extravascular lung water in patients undergoing CPB,^1^ no excess of pulmonary tissue was observed postoperatively, as attested by the lack of increase in lung weight. It is highly likely that the lack of increase in postoperative tissue volume resulted from 2 mechanisms: 1) A decrease in circulating pulmonary blood volume and flow after the closure of the left-right shunt; and 2) The use of hemofiltration during CPB controlling the increase in extravascular lung water.

### Effects of positive end-expiratory pressure on lung aeration loss

After application of PEEP, pulmonary gas volume increased progressively, restoring the amount of normally aerated parenchyma to preoperative values and reducing the amount poorly aerated lung parenchyma at PEEP 10 cm H_2_O but failed to re-expand the collapsed regions. The distribution of pressure-induced increase in gas volume within the lung parenchyma is mainly determined by regional lung compliance,^8^ which depends on intrinsic elasticity and the number of open pulmonary units in each a given region.^25^ Moderate PEEP levels are reported to improve oxygenation and reduce time to extubation,^26^ but the airway pressures required to open collapsed lung units were likely higher than 20 to 25 cm H_2_O, the range of peak inspiratory pressure used in the present study.^27^ At least 2 mechanisms may explain why very high pressure are required to re-expand collapsed lung regions in children undergoing surgical repair of congenital heart disease. First, the chronic high pulmonary blood flow resulting from left-to-right intra-cardiac shunt causes interstitial edema, stiffening of the pulmonary interstitium, arterial vasodilation, increase in capillary bed and increase in pulmonary blood volume. These pathological abnormalities, which are not immediately reversible following surgery, increase lung elastance and act as potent counterforces against positive intrathoracic pressure (PEEP or recruitment maneuver), supposed to re-open collapsed lung regions.^28^ Second, children with congenital heart disease have marked cardiac enlargement, the heart laying over and compressing lower lobes. The resulting superimposed pressure may explain why only high intrathoracic pressure may keep the lung open in the postoperative period. In adults submitted to cardiac surgery under general anesthesia, recruitment maneuvers using airway pressures of 40 cm H_2_O have been shown to effectively recruit lung collapsed areas.^29^ In pediatric population under 2 years of age, the potential benefit of such maneuvers remains unknown. The possibility of detrimental effects in a population of children with not fully developed alveolar saccules and airways cannot be ruled out and raise the important issue of risk to benefit ratio.^30^ One possible alternative strategy to cope with dependent lung collapse could be prone positioning that eliminates lung compression by the heart.^24^

In conclusion, surgical closure of heart defects under cardiopulmonary bypass induces a substantial loss of aeration. A positive end-expiratory pressure of 10 cm H_2_O reverses partially aeration loss by recruiting poorly aerated lung regions but fails to decrease the amount of non-aerated parenchyma. Further studies are necessary to evaluate possible strategies, such as individualized PEEP titration, recruitment maneuvers, or adjunctive therapies, to restore normal gas distribution and reverse postoperative atelectasis within the lungs of small children undergoing surgical repair of congenital heart disease.

## Declaration of competing interest

The authors declare no conflicts of interest.
